# High-intensity, whole-body exercise improves blood pressure control in individuals with spinal cord injury: A prospective randomized controlled trial

**DOI:** 10.1371/journal.pone.0247576

**Published:** 2021-03-04

**Authors:** Ryan Solinsky, Adina Draghici, Jason W. Hamner, Rich Goldstein, J. Andrew Taylor

**Affiliations:** 1 Cardiovascular Research Lab, Spaulding Rehabilitation Hospital, Cambridge, Massachusetts, United States of America; 2 Department of Physical Medicine & Rehabilitation, Harvard Medical School, Boston, Massachusetts, United States of America; 3 Spaulding Research Institute, Boston, Massachusetts, United States of America; University of Maryland School of Medicine, UNITED STATES

## Abstract

Blood pressure regulation following spinal cord injury (SCI) is often compromised due to impaired vascular sympathetic control, leading to increased reliance on cardiovagal baroreflex sensitivity to maintain pressure. Whole-body exercise improves cardiovagal baroreflex sensitivity in uninjured individuals, though has not been explored in those with SCI. Our objective was to determine changes in cardiovagal baroreflex sensitivity following 6 months of high-intensity, whole-body exercise in individuals with SCI compared to lower-intensity, arms only exercise, or waitlist. This randomized controlled trial recruited individuals with SCI aged 18–40 years old. Sixty-one individuals were randomized, with 38 completing at least one cardiovagal baroreflex sensitivity assessment. Whole-body exercise was performed with hybrid functional electrical stimulation rowing prescribed as two to three times per week, for 30–60 minutes with a target heart rate of >75% of maximum. The arms only exercise group performed upper body rowing exercise with the same prescription as whole-body exercise. Waitlist controls were not enrolled in any explicit training regimen. After 6 months, those in arms only exercise or waitlist crossed over to whole-body exercise. Cardiovagal baroreflex sensitivity was assessed via the neck suction technique at baseline and at three-month intervals thereafter. Intention to treat analysis with a structured equation model demonstrated no significant effect of waitlist control or arms only exercise on cardiovagal baroreflex sensitivity. Whole-body exercise significantly improved cardiovagal baroreflex sensitivity at 6 months for those initially randomized (p = 0.03), as well as those who crossed over from arms only exercise or waitlist control (p = 0.03 for each). However, amount of exercise performed and aerobic gains (VO_2max_) each poorly correlated with increases in cardiovagal baroreflex sensitivity (R^2^<0.15). In post-hoc analyses, individuals with paraplegia made significantly greater gains in baroreflex sensitivity compared to those with tetraplegia (p = 0.02), though gains within this group were again poorly correlated to gains in aerobic capacity. Clinicaltrials.gov number NCT02139436.

## Introduction

Blood pressure regulation is often compromised following spinal cord injury (SCI), clinically manifesting as orthostatic hypotension and autonomic dysreflexia [1,2]. Blood pressure is exquisitely regulated on a beat-to-beat basis through the cardiovagal baroreflex [3,4], wherein acute changes in blood pressure are sensed by the carotid and aortic baroreceptors and buffered by vagally-mediated changes in heart rate. The sensitivity of this cardiovagal reflex arc, measured as the magnitude of increase in time between heart beats in response to a given increase in systolic pressure, is commonly decreased following SCI [5,6]. Improvements in the ability to counter-regulate blood pressure changes would be especially important after SCI where blood pressure may surge during conditions such as autonomic dysreflexia. In addition to compensating during autonomic dysreflexia, cardiovagal baroreflex sensitivity has clear clinical implications for cardiovascular health. Sensitivity below 3.0 ms/mmHg is associated with significantly greater cardiovascular mortality [7]. This is particularly relevant for those with chronic SCI in whom cardiovascular disease is the leading source of death and occurs at nearly three times that of the general population [8,9].

SCI often results in loss of sympathetic control below the neurological level of injury. The physiological implications of this are twofold. First, with limited ability to engage inhibition of sympathetic activity to the vasculature, those with SCI would rely more heavily on cardiovagal baroreflex control for blood pressure homeostasis. For example, during episodes of autonomic dysreflexia, vasoconstriction leads to systolic blood pressure (SBP) increases of over 20 mmHg which can result in profound bradycardia [2,10]. Secondly, with loss of sympathetic control, classic measures of baroreflex sensitivity that require systemic vascular sympathetic engagement, such as vasoactive drugs or Valsalva’s maneuver, may not provide accurate estimates [4]. Valsalva’s maneuver provides a potent example; 80% of those with SCI above T6 demonstrate no phase II pressure stabilization due to lack of sufficient sympathetic vasoconstrictor activity [11]. This renders this common test, with well characterized phases and techniques to calculate baroreflex sensitivity, inadequate for such purposes. Though appraising cardiovagal baroreflex sensitivity could have significant import to understanding how to improve blood pressure control after SCI, the loss of systemic sympathetic regulation makes accurate assessment intrinsically more difficult.

In individuals without SCI, prior work has demonstrated that aerobic exercise training improves baroreflex sensitivity [3,12,13] and that the magnitude of improvement is related to the intensity of exercise performed and commensurate with gains in aerobic capacity (via VO_2max_) [3,12]. However, this has not been explored in those with SCI and the ability to perform high-intensity, whole-body exercise is often limited by paralysis in these individuals. Hybrid-functional electrical stimulation (FES) rowing provides one option to recruit greater muscle mass for whole-body exercise in this population, and as such, may have unique potential to improve blood pressure regulation after SCI. Given the crucial importance of blood pressure regulation in these individuals, our objective was to determine the effects of 6 months of high-intensity, whole-body exercise compared to standard of care on cardiovagal baroreflex sensitivity in individuals with SCI. To reliably assess cardiovagal baroreflex sensitivity, we employed a neck suction technique which directly engages the carotid baroreceptors and does not require vascular sympathetic engagement. We then conducted a randomized controlled trial of 6 months of exercise with crossover design. Our results suggest that high-intensity, whole-body exercise can improve blood pressure regulation, but occurs primarily in individuals with paraplegia as opposed to those with tetraplegia. Importantly, in contrast to uninjured individuals, baroreflex sensitivity improvements are poorly correlated to aerobic gains following intense exercise training.

## Materials and methods

### Trial design

A parallel randomized controlled trial was undertaken to assess the ability of 6 months of whole-body exercise to alter baroreflex sensitivity following subacute spinal cord injury. Individuals were randomized to either 6 months of whole-body exercise, consisting of hybrid- FES assisted rowing, an arms only exercise intervention, or waitlist control. This protocol was approved by the Mass General Brigham Institutional Review Board (Protocol #2013P000604).

### Participants

Individuals were eligible for inclusion in this randomized controlled trial if they were aged 18–40 with traumatic SCI, American Spinal Injury Association Impairment Scale (AIS) grades A-C [14], and neurological levels of injury (NLI) C1-T10 (Table 1). Age was restricted to minimize potential confounding effects of aging on cardiovagal baroreflex sensitivity [15]. All individuals had ≥4/5 strength in at least one C5 myotome (elbow flexors), allowing them to utilize the needed exercise equipment and make potential gains in aerobic capacity from whole-body exercise. All individuals were at least 3 months post-injury and had been discharged to the community from inpatient rehabilitation prior to enrollment. Recruitment was targeted at individuals who were less than 24 months post-injury to limit the effects of extended detraining. Individuals were screened for medical conditions that precluded exercise training [16]. Potential participants with uncontrolled autonomic dysreflexia were further excluded to ensure this did not interfere with the exercise program. All individuals provided written informed consent prior to enrollment in this IRB approved study. The authors confirm that all ongoing and related trials for this intervention are registered (NCT02139436).

**Table 1:** Demographics for enrolled individuals with spinal cord injuries per initial group assignments.

		Waitlist Controls (n = 11)	Arms Only Exercise (n = 11)	Whole-Body Exercise (n = 16)
**Age (years)**		25.7 ± 1.7	28.9 ± 1.8	30.8 ± 1.3
**Sex**		1F/10M	1F/10M	2F/14M
**NLI**	**C1-C4**	4 (36.4%)	1 (9.1%)	3 (18.8%)
	**C5-C8**	3 (27.3%)	3 (27.3%)	7 (43.4%)
	**T1-T6**	3 (27.3%)	4 (36.4%)	3 (18.8%)
	**T7-T10**	1 (9.1%)	3 (27.3%)	3 (18.8%)
**AIS**	**A**	8 (72.7%)	6 (54.5%)	8 (50.0%)
	**B**	1 (9.1%)	2 (18.2%)	6 (37.5%)
	**C**	2 (18.2%)	3 (27.3%)	2 (12.5%)
**Chronicity of SCI (months)**		10.6 ± 0.9	8.4 ± 1.0	12.4 ± 1.5

Whole-body exercise was performed with hybrid functional electrical stimulation rowing. No significant differences were found between groups. Mean values presented ± SE. NLI = Neurological Level of Injury, AIS = American Spinal Injury Association Impairment Scale.

### Interventions

For the whole-body exercise group, individuals were enrolled in an intensive hybrid-FES assisted rowing program for 30–60 minute sessions, 2–3 days a week for 6 months. Individuals were instructed to maintain wattage output to achieve >75% of exercise-tested maximal heart rate, based on previous findings suggesting this level of intensity is required to achieve meaningful gains in aerobic fitness after SCI [17]. Exercise intensity was adjusted at 3 months based on interim VO_2 max_ testing. Full details of this hybrid-FES rowing protocol have been described previously [17]. Individuals in the arms only exercise group were enrolled in arms only rowing with an exercise prescription that matched that of the whole-body exercise group. Individuals in the waitlist group were not provided structured exercise.

### Outcomes

This study addressing changes in baroreflex sensitivity was the primary endpoint of a broader study looking at multiple exercise endpoints (NCT02139436). Individuals in the arms only exercise and waitlist groups had baseline and 6-month VO_2 max_ testing completed using an arms only rowing to quantify changes in aerobic fitness over this period. Those in the whole-body exercise group had baseline and 6-month VO_2 max_ testing completed with hybrid FES-assisted rowing, their training modality. Total amount of exercise for each individual was quantified by distance rowed over the 6-month period, average percentage of maximum heart rate achieved per exercise session, and total time logged on the rowing machine. Given known inaccuracies in generalized age-predicted maximal heart rates following SCI, individualized values obtained during baseline maximum exercise testing were used. To assess any potential contribution of NLI, clinical testing with the International Standards for Neurological Classification of Spinal Cord Injury (ISNCSCI) exam [14] was completed by a trained SCI medicine physician at baseline.

Cardiovagal baroreflex sensitivity was assessed in the laboratory at baseline and at 3-month intervals for all groups throughout the study. Individuals were requested not to engage in vigorous activity or consume alcohol or caffeine for 24 hours prior to all assessments. All testing was completed in the morning following a 12 hour overnight fast. Individuals were instrumented with a 5-lead EKG, finger photoplethysmography (Finaprep, Ohmeda Medical) for beat-to-beat blood pressure (calibrated against an automated brachial cuff, Dinamap Dash 2000, GE), and nasal canula to monitor respiration via expiratory CO_2_ measurements. Five minutes of supine rest data were collected prior to any testing to ensure an adequate baseline and compare any changes in mean resting pressure. Carotid baroreceptor-cardiac reflex responses were elicited during held expiration by application of external neck suction via a malleable chamber that enclosed the anterior neck from chin to clavicle (Fig 2A). Per well-established convention [18,19], individuals held a normal end-expiratory volume and the custom collar provided external neck suction over the carotid baroreceptors at 10, 20, 30, or 40 mmHg over a period of four cardiac cycles (Fig 2B). Each stimulus was repeated five times with at least 30 seconds between each pressure application. In this way, a measured amount of carotid distending pressure (mmHg) at the baroreceptors was applied. By incorporating this applied distending pressure to the pre-neck suction systolic pressure, an accurate pressure experienced by the carotid baroreceptors can be calculated. The corresponding changes in R-R interval experienced due to this change in pressure were recorded, with all data digitized and stored at 1kHz using PowerLab software (PowerLab, ADInstruments) for analysis.

### Sample size

An enrollment target of 60 individuals with SCI was calculated to achieve adequate statistical power (see supplemental information).

### Randomization

**Fig 1:**
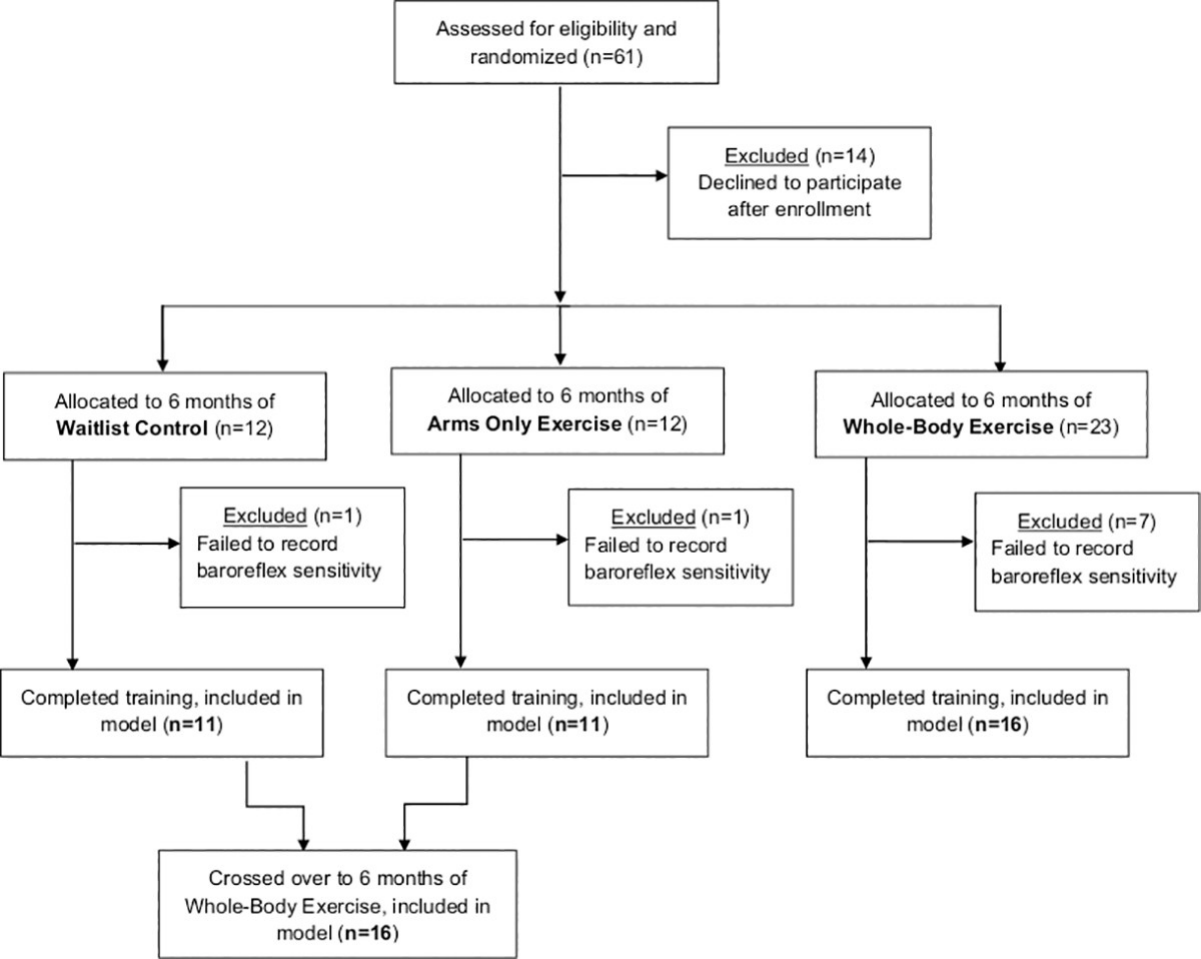
Study participant inclusion flow chart.

Simple randomization was performed if individuals met our narrow inclusion criteria. Allocation ratio between the groups was 2:1:1 with individuals in the arms only exercise or waitlist control groups intended to cross over to the whole-body exercise group after 6 months (Fig 1). Allocation sequence was developed by the study statistician and there was full concealment from the participant and investigators until participants were enrolled. Enrollment was unblinded and performed by study coordinators not involved with data analysis. Given underlying secondary medical complications of spinal cord injury, assignments to waitlist group were, at times, extended past 6 months. For example, if someone developed a pressure injury and was unable to begin whole-body exercise once the waitlist period had ended, this waitlist period was extended until they were able to participate in the cross over.

### Statistical methods

**Fig 2:**
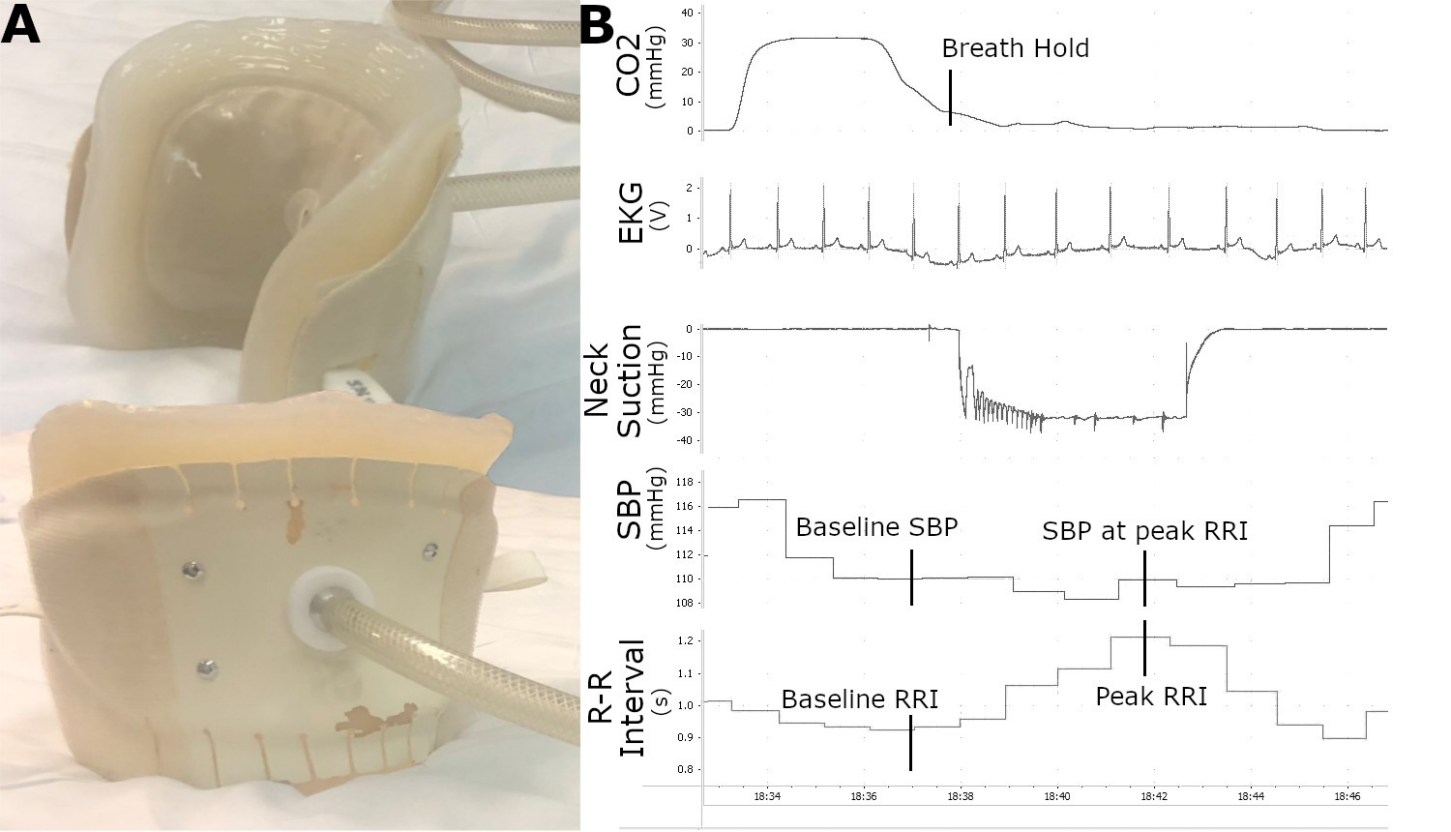
A) Neck suction collar device which is applied over the anterior neck. Top image illustrating the inner portion where suction is applied, bottom image illustrating suction hose attachment; B) Example of key baroreflex sensitivity calculation measurements utilizing neck suction collar. Please see *Statistical Methods* subsection for details on calculating baroreflex sensitivity from these measurements.

Cardiovagal baroreflex sensitivity was calculated as the change in R-R interval (ms) per change in SBP (mmHg) at the carotid baroreceptor and was averaged for each individual over the range of pressures applied with neck suction. R-R interval change was measured as the difference from the beat prior to neck suction initiation to maximum R-R interval lengthening during the neck suction stimulus. Per convention, change in SBP at the carotid baroreceptors was derived from the sum of SBP at peak R-R interval response and the magnitude of neck suction, minus the SBP immediately prior to neck suction [20,21] (Fig 2). Trials of neck suction where an expiratory breath hold was not maintained were excluded from analysis due to potential for confounding effects of respiration. Baseline demographics were assessed for normality and compared using one-way ANOVAs. *A priori* power calculations demonstrated adequate power for detecting changes between baseline and 6-month baroreflex sensitivities (our primary endpoint) with an alpha of 0.05 with a total of 60 enrolled individuals, allowing for 15% dropout.

An intention to treat analysis was completed with all available data for baroreflex sensitivity for the three intervention groups. A structural equation model, utilizing full information maximum likelihood was created with STATA (StataCorp. v16). This model assumes baroreflex sensitivity values are missing at random, based conditionally upon group assignment. Observed effect sizes were calculated compared to baseline cardiovagal baroreflex sensitivities in three-month intervals, depending upon group assignment. Akaike’s information and Bayesian information criteria were calculated to assess relative model fit. Post-hoc supplemental analyses were completed in STATA to assess the role of intensity of whole-body exercise on cardiovagal baroreflex sensitivity change. Pearson’s product-moment correlation coefficient was calculated to determine associations between baroreflex sensitivity and potential influencing factors including age, NLI, AIS, and chronicity of injury. Changes in resting R-R interval and mean blood pressure during the 5-minute baseline period were further compared. Within the whole-body exercise group, individuals with paraplegia were further compared to those with tetraplegia using heteroscedastic t-tests for baseline variable and two-way ANOVA for comparisons of VO_2max_, R-R interval, baroreflex sensitivity, and resting blood pressure changes. A p-value of <0.05 was deemed as statistically significant. Data are presented as mean ± standard error of the mean (SE).

## Results

Sixty-one individuals met inclusion criteria, were enrolled, and randomized, with 47 participating in exercise or the waitlist, and 38 completing at least one assessment of cardiovagal baroreflex sensitivity from May 2014 to completion on May 2019 (Fig 1). This included 11 individuals in the waitlist group, 11 individuals in the arms only exercise group, and 16 individuals in the whole-body exercise group. Within these 38 individuals, 123 testing sessions were recoded. Accurate cardiovagal baroreflex sensitivities were able to be calculated for 66.7% of the testing sessions, with 41 excluded (35 due to incomplete breath hold during baroreflex testing, 3 due to lack of R-R interval change, and 3 due to recording equipment malfunction). Comparison between demographics for individuals in the waitlist, arms only exercise, and whole-body exercise groups demonstrated no significant difference in age (F = 2.79, p = 0.07), sex (F = 0.05, p = 0.95), NLI (F = 1.99, p = 0.15), AIS (F = 0.32, p = 0.73), or chronicity of injury (F = 2.54, p = 0.09; Table 1). Chronicity of injury was not correlated with baseline cardiovagal baroreflex sensitivity (R^2^ = 0.03).

At baseline, there were so significant differences between groups for VO_2max_ (F = 0.56, p = 0.58), resting R-R interval (F = 0.14, p = 0.87), cardiovagal baroreflex sensitivity (F = 1.81, p = 0.19), resting systolic (F = 0.45, p = 0.64), or diastolic blood pressure (F = 1.90, p = 0.16; Table 2). Individuals in the waitlist group did not have structured exercise. The individuals who completed arms only exercise logged a mean of 108.9 ± 22.5 km, with heart rate averaging 82.6 ± 0.8% of maximum over 13.9 ± 2.4 hours of exercise during the 6 months of training. In the whole-body exercise group with hybrid-FES, individuals rowed a mean of 131.7 ± 13.4 km, with heart rate averaging 81.8 ± 0.2% of maximum over 16.9 ± 1.6 hours of exercise. There were no related adverse events in any of the group assignments for included individuals.

**Table 2:** Changes in key metrics over 6 months participation in each group.

	Waitlist Controls		Arms Only Exercise		Whole-Body Exercise	
	*Baseline*	*6 months*	*Baseline*	*6 months*	*Baseline*	*6 months*
**VO**_**2max**_ **(ml/kg/min)**	14.6 ± 3.4	13.9 ± 2.5	18.5 ± 3.7	18.2 ± 4.4	17.1 ± 1.2	18.9 ± 1.5*
**Resting R-R Interval (ms)**	988 ± 62	1,079 ± 90	1,030 ± 110	1,042 ± 160	977 ± 47	972 ± 45
**Baroreflex Sensitivity (ms/mmHg)**	4.1 ± 1.0	3.7 ± 1.0	3.2 ± 0.8	2.8 ± 1.0	2.5 ± 0.4	3.5 ± 0.7*
**Resting Systolic Blood Pressure (mmHg)**	116.2 ± 3.2	119.0 ± 3.8	121.9 ± 3.8	117.1 ± 2.8*	116.0 ± 3.3	118.0 ± 3.4
**Resting Diastolic Blood Pressure (mmHg)**	58.3 ± 3.8	60.9 ± 4.6	70.1 ± 4.1	66.9 ± 5.7	64.9 ± 2.7	66.3 ± 3.1

Mean values presented ± SE.

*Change of p<0.05.

In the waitlist group, VO_2max_ was unchanged at 6-month testing (mean decline of 0.8 ± 1.6 ml/kg/min, p = 0.66). In the arms only exercise group, VO_2max_ was also unchanged (mean decline of 0.3 ± 1.1 ml/kg/min, p = 0.71). In the whole-body exercise group, mean VO_2max_ increased after 6 months of training (mean increase of 1.7 ± 0.7 ml/kg/min, p = 0.03). Resting R-R interval did not significantly change within any of study groups in comparison to baseline (p>0.22, Table 2). Mean resting systolic and diastolic blood pressures were not significantly changed by 6 months of waitlist or whole-body exercise (p>0.42, Table 2). In the arms only exercise group, mean resting systolic pressure decreased by 4.7 mmHg over the 6 months (p = 0.02), while diastolic pressure was unchanged (p = 0.60).

**Fig 3:**
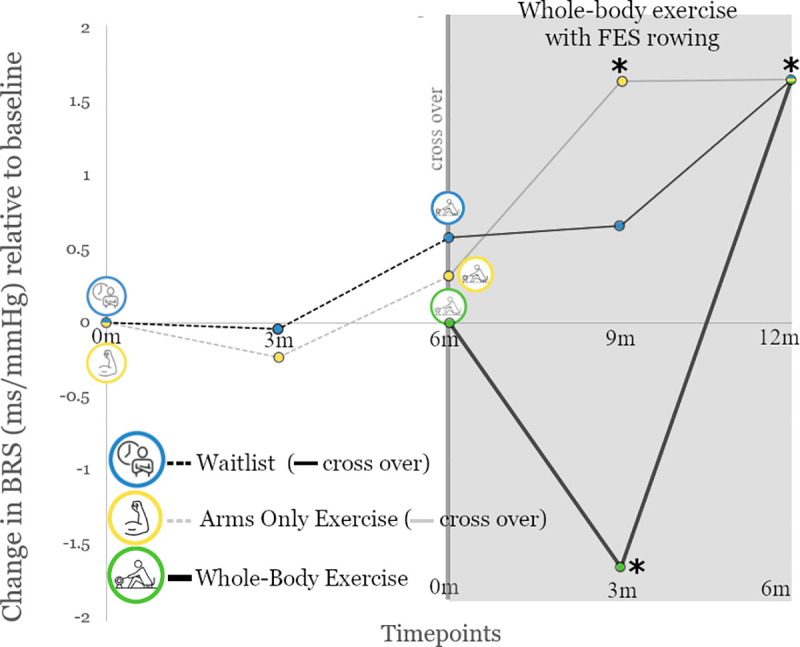
Mean changes in baroreflex sensitivity. Change from baseline baroreflex sensitivity (BRS) for waitlist (n = 11), arms only exercise (n = 11), and whole-body exercise with hybrid-functional electrical stimulation (FES) rowing (n = 16) using results from the structural equation model. Note, after six months, individuals in waitlist and arms only exercise groups crossed over into whole-body exercise for an additional six months of training utilizing hybrid-FES rowing. As relative changes, all values start at baseline values of zero. The final timepoint at 6 months of hybrid-FES rowing converges at a single value due to the structural equation model quantifying changes between marked time points for each intervention, with the final time point identifying changes from 3 to 6 months of hybrid-FES rowing for all. * = results where p<0.05.

The structural equation model, which incorporated all available data at 3-month intervals, demonstrated a strong relative fit according to Akaike’s and Bayes information criteria (352.7 and 374.4). The model demonstrated no significant changes in cardiovagal baroreflex sensitivity from baseline to 3-month, or 3-month to 6-month time points for the waitlist or arms only exercise groups (Fig 3). When these individuals crossed over to whole-body exercise, significant increases in baroreflex sensitivity were demonstrated at 3 months for the waitlist group (p = 0.04) and at 6 months for both groups (p = 0.03). From baseline, individuals randomized into whole-body exercise demonstrated a statistically significant initial decline in baroreflex sensitivity at 3 months (p<0.001) which subsequently increased at 6 months relative to baseline (p = 0.03). As this model assumes that baroreflex sensitivity is constant by comparing all values to the baseline mean, post-hoc analyses were performed on 3-month changes within the whole-body exercise group. Of the 10 individuals within this group who had both baseline and 3-month baroreflex sensitivities recorded, mean initial baroreflex sensitivity was 3.35 ± 0.62 ms/mmHg and decreased to a mean of 3.27 ± 0.51 ms/mmHg at 3-months (p = 0.89), indicating that this finding in the structural equation model may be an artifact rooted in a lower number of 3-month timepoint measures.

Contrary to previous findings in uninjured populations, the magnitude of change in cardiovagal baroreflex sensitivity was minimally correlated to any measure of amount of whole-body exercise completed (kilometers rowed, R^2^ = 0.01, percentage maximum heart rate averaged, R^2^ = 0.06, average minutes rowing per week, R^2^ = 0.15, or VO_2 max_ change, R^2^ = 0.02). Further, within this whole-body exercise cohort, baseline demographic characteristics such as age (R^2^ = 0.01), NLI (R^2^ = 0.21), AIS (R^2^ = 0.10), and chronicity of SCI (R^2^ = 0.03) were all minimally correlated to change in cardiovagal baroreflex sensitivity.

The 16 individuals who completed 6 months of whole-body exercise and had full baseline and endpoint baroreflex recordings were further dichotomized to either paraplegia (n = 6) or tetraplegia (n = 10). Individuals with paraplegia had a higher baseline VO_2max_ (p = 0.006), with no differences in resting R-R interval (p = 0.34), cardiovagal baroreflex sensitivity (p = 0.16), resting systolic (p = 0.07) or diastolic pressure at baseline (p = 0.44, Table 3). Despite engaging in similar levels of exercise by all metrics (Table 3) and having similar VO_2max_ increases (F = 0.03, p = 0.85) and resting R-R interval changes (F = 0.01, p = 0.91), individuals with paraplegia demonstrated significantly greater increases in baroreflex sensitivity with whole-body exercise (F = 5.61, p = 0.02). This increase in baroreflex sensitivity in individuals with paraplegia was again poorly correlated to measures of exercise performed (VO_2max_ change, R^2^ = 0.003, percent maximum heart rate, R^2^ = 0.05, minutes rowed, R^2^ = 0.10, meters rowed, R^2^ = 0.007). Resting systolic and diastolic pressures were not significantly altered by 6 months of whole-body exercise (F = 0.28, p = 0.60 and F = 0.54, p = 0.47 respectively).

**Table 3:** Effects of 6 months of whole-body exercise with hybrid functional electrical stimulation rowing for individuals with paraplegia and tetraplegia.

	**Individuals with paraplegia (n = 6)**		**Individuals with tetraplegia (n = 10)**		
**Kilometers rowed**	140.3 ± 18.2		126.1 ± 18.9		p = 0.61
**Percent maximum heart rate averaged**	83.8 ± 4.1%		80.6 ± 2.2%		p = 0.66
**Minutes rowed/week**	39.7 ± 6.6		34.8 ± 4.8		p = 0.55
	***Baseline***	***6 months***	***Baseline***	***6 months***	
**VO**_**2max**_ **(ml/kg/min)**	21.4 ± 1.6	22.9 ± 2.2	14.6 ± 1.1	16.5 ± 1.6	F = 0.03, p = 0.85
**R-R interval (ms)**	924 ± 65	912 ± 31	1,017 ± 66	1,017 ± 73	F = 0.01, p = 0.91
**Baroreflex sensitivity (ms/mmHg)**	3.28 ± 0.7	5.89 ± 1.3	2.04 ± 0.5	2.14 ± 0.4	F = 5.61, p = 0.02
**Resting Systolic Blood Pressure (mmHg)**	124.4 ± 4.0	123.7 ± 6.6	112.4 ± 4.2	115.5 ± 4.0	F = 0.28, p = 0.60
**Resting Diastolic Blood Pressure (mmHg)**	68.8 ± 5.3	73.1 ± 5.9	52.2 ± 3.0	57.0 ± 3.5	F = 0.54, p = 0.47

Mean values presented ± SE.

## Discussion

After SCI, whole-body exercise with hybrid-FES rowing demonstrates significant improvements in blood pressure regulation as seen by increases in cardiovagal baroreflex sensitivity. No significant improvements were seen with either the absence of a structured exercise program (waitlist controls) or arms only exercise, a common form of exercise following SCI. Interestingly, when these individuals crossed over into whole-body exercise, those initially in the waitlist did not have significant improvements in baroreflex sensitivity at 3 months, whereas those in the arms only group did. This may speak to a needed level of pretraining which much occur prior to autonomic benefits. This idea is further supported by individuals who were randomized directly into the whole-body exercise group, where 3 months of intervention were ineffective. The degree of relative improvement in cardiovagal baroreflex sensitivity (+41.6%) with whole-body exercise following SCI is greater than that which has previously been described following exercise in uninjured individuals (25%, [12]). This is likely due, in part, to the low baseline baroreflex sensitivity present in that many individuals with SCI. However, contrary to gains with exercise in uninjured individuals [3], increases in cardiovagal baroreflex sensitivity were poorly correlated with the amount of training or gains in aerobic capacity. Further efforts to identify the optimal demographic for this intervention following SCI, be it by age, injury severity or chronicity, did not yield any strong clinical insights. This may not be surprising, as our age range was restricted to minimize confounders, and most clinically utilized assessments of SCI rely on motor/sensory examination and largely ignore autonomic involvement. Future detailed autonomic assessments in this population may better identify those who will benefit from whole-body exercise.

Our structural equation model, which demonstrated significant improvements in cardiovagal baroreflex sensitivity, was well fit to the data (as seen by high Akaike’s information and Bayesian information criteria). It is, however, based upon the assumption that within our study window, cardiovagal baroreflex sensitivity does not change relative to baseline. This assumption is supported by our data; individuals in the waitlist control group had non-significant changes over the first 6 months, prior to potential cross over to whole-body exercise. Further, across all individuals with SCI, we found that baseline baroreflex sensitivity was poorly correlated to chronicity of injury. It is unclear, however, how baroreflex sensitivity changes after the first two years of SCI, and it is reasonable to assume that ongoing deconditioning may lead to significant declines [22,23]. As such, extrapolation of these results to more chronic SCI may not be appropriate.

Of note, mean resting systolic and diastolic pressures were unchanged by both waitlist and whole-body exercise. Given our small sample size, this lack of significant mean resting pressure change is not unexpected. In large meta-analyses of over 2,000 individuals, decreases of 1 to 2 mmHg in systolic pressure have been quantified in normotensive individuals with prolonged aerobic exercise [24]. Similar effects are possible after SCI, though our study was not powered to detect these differences. Interestingly, mean resting systolic pressure was decreased by 4 mmHg with the arms only exercise intervention. It is unclear the clinical significance of this decrease or if this represents regression toward a mean value of systolic pressure. Importantly, this study was designed to assess how individuals with SCI buffer changes in blood pressure, a clinically important metric due high rates of autonomic dysreflexia [25]. As resting blood pressure is more closely related to basal sympathetic tone, induced changes with interventions may have increased variability in this population [26]. Blood pressure control is extremely important after SCI, and further directed study is needed.

Within individuals with SCI who participated in 6 months of whole-body exercise, individuals with paraplegia had a significantly greater increase in cardiovagal baroreflex sensitivity compared to those with tetraplegia, despite similar amounts exercise performed and aerobic gains. While limited by numbers, future well-powered studies aimed at deciphering the role of cardiac sympathetic innervation and blood volume regulation within individuals with high vs low level paraplegia would be helpful. As a whole, individuals with paraplegia demonstrated a higher baseline VO_2max_, which may signal that they not only are able to recruit more muscle, but that they are able to generate greater blood pressure changes with exercise and potentially have less baseline arterial stiffness. This combination may contribute to the increased gains in baroreflex sensitivity seen following paraplegia and not tetraplegia. Correspondingly, individuals with tetraplegia may have baseline arterial stiffening, translating to lesser increases in blood pressure during exercise. Hence, the exercise pressor stimulus is smaller and may therefore induce smaller baroreflex adaptations. While limited in power by smaller numbers in this sub-analysis, this is still clinically sobering. Individuals with tetraplegia are at the highest risk for cardiovascular disease and the effects of autonomic dysregulation, so further research into how individuals with tetraplegia may perform exercise that can mitigate these risks is desperately needed.

While this study was not designed to draw direct clinical implications of improvements in blood pressure regulation, it is difficult to argue that such substantial improvements are not positive. In addition to autonomic dysreflexia and orthostatic hypotension, dysregulated blood pressure after SCI has further been proposed to contribute to cardiovascular disease through labile blood pressures [27], cognitive dysfunction from chronic orthostatic hypotension [28], or predisposition to pressure injuries from altered skin perfusion [29]. Despite pharmacologic agents often being effective for managing blood pressure during acute SCI-related autonomic crises [30,31], there is little evidence to suggest these medications play a more permanent role in altering central autonomic pathways. By eclipsing the 3.0ms/mmHg sensitivity threshold found by LaRovere [7] as an important marker for increased risk of cardiovascular mortality, this whole-body exercise has the potential to improve cardiac risk profiles, especially for those with paraplegia.

### Limitations

R-R interval responses are determined by a complex interplay of sympathetic and parasympathetic signals, an interplay made even more complex by varied levels of spared supraspinal autonomic signals and corrupted circuits below the level of lesion. Baseline levels of autonomic dysregulation can occur after SCI, and thus it is unclear how balanced our three sample groups are with respect to “autonomic completeness.” If an individual had greater baseline vagal tone, their potential responses to neck suction may be accentuated. This concept, termed *accentuated antagonism*, effectively changes the starting position for potential baroreflex sensitivity changes [32]. Similarly, vagal responsiveness can be blunted by increases in sympathetic tone which may be variable within the same individual with SCI between testing sessions (increased for example on days between bowel programs). While the neck suction technique eliminates the direct influence of sympathetic tone during the testing period, it is unclear how sustained increases in this tone may alter the vagal balance prior to testing. Additionally, with concern to balance within our study groups, individuals were randomized if meeting the narrow inclusion criteria alone. This introduces the potential that sub-strata (as illustrated in Table 1) may be unequally balanced. However, given our results that all groups experienced significant increases in baroreflex sensitivity following 6 months of whole-body exercise, including those who crossed over from waitlist or arms only exercise, we feel confident that the observed effects are real and not due to an artifact from unequal randomization. Further, many baroreflex testing sessions (33%) were unable to generate meaningful data, driven in large part by inability to maintain breath hold during the neck suction. This raises the potential that individuals who were unable to time breath holds, be it for respiratory insufficiency or cognitive reasons, were underrepresented in our study. Finally, our study had purposefully narrow inclusion criteria with respect to participant age and time since injury. This was designed to limit confounders and identify potential effect in the subgroup thought most likely to experience it. However, broad generalizability is limited by this approach, and further study is needed to see if these gains in baroreflex sensitivity translate to wider encompassing population of individuals with SCI.

## Conclusions

For individuals with spinal cord injury, 6 months of high-intensity, whole-body exercise with hybrid functional electrical stimulation rowing results in significant improvements in cardiovagal baroreflex sensitivity.

## Supporting information

S1 Checklist Study CONSORT diagram. (DOC)

S1 File Detailed IRB protocol. (DOCX)

S2 File Full data available at *Harvard dataverse* https://doi.org/10.7910/DVN/JJGTYM. (DOCX)

## References

[1] Garstang SV, Miller-Smith SA. Autonomic nervous system dysfunction after spinal cord injury. Physical medicine and rehabilitation clinics of North America. 2007 5 1;18(2):275–96. 10.1016/j.pmr.2007.02.003 17543773

[2] Karlsson AK. Autonomic dysreflexia. Spinal cord. 1999 6;37(6):383–91. 10.1038/sj.sc.3100867 10432257

[3] Deley G, Picard G, Taylor JA. Arterial baroreflex control of cardiac vagal outflow in older individuals can be enhanced by aerobic exercise training. Hypertension. 2009 5 1;53(5):826–32. 10.1161/HYPERTENSIONAHA.109.130039 19332656 PMC2696114

[4] Draghici AE, Taylor JA. Baroreflex autonomic control in human spinal cord injury: physiology, measurement, and potential alterations. Autonomic Neuroscience. 2018 1 1;209:37–42. 10.1016/j.autneu.2017.08.007 28844537

[5] Koh J, Brown TE, Beightol LA, Ha CY, Eckberg DL. Human autonomic rhythms: vagal cardiac mechanisms in tetraplegic subjects. The Journal of physiology. 1994 2 1;474(3):483–95. 10.1113/jphysiol.1994.sp020039 8014908 PMC1160339

[6] Convertino VA, Adams WC, Shea JD, Thompson CA, Hoffler GW. Impairment of carotid-cardiac vagal baroreflex in wheelchair-dependent quadriplegics. American Journal of Physiology-Regulatory, Integrative and Comparative Physiology. 1991 3 1;260(3):R576–80. 10.1152/ajpregu.1991.260.3.R576 2001007

[7] La Rovere MT, Bigger JT Jr, Marcus FI, Mortara A, Schwartz PJ, ATRAMI (Autonomic Tone and Reflexes After Myocardial Infarction) Investigators. Baroreflex sensitivity and heart-rate variability in prediction of total cardiac mortality after myocardial infarction. The Lancet. 1998 2 14;351(9101):478–84. 10.1016/s0140-6736(97)11144-8 9482439

[8] Cragg JJ, Noonan VK, Krassioukov A, Borisoff J. Cardiovascular disease and spinal cord injury: results from a national population health survey. Neurology. 2013 8 20;81(8):723–8. 10.1212/WNL.0b013e3182a1aa68 23884034 PMC3776463

[9] Myers J, Lee M, Kiratli J. Cardiovascular disease in spinal cord injury: an overview of prevalence, risk, evaluation, and management. American journal of physical medicine & rehabilitation. 2007 2 1;86(2):142–52. 17251696 10.1097/PHM.0b013e31802f0247

[10] Solinsky R, Kirshblum SC, Burns SP. Exploring detailed characteristics of autonomic dysreflexia. The journal of spinal cord medicine. 2018 9 3;41(5):549–55. 10.1080/10790268.2017.1360434 28784041 PMC6127514

[11] Berger MJ, Kimpinski K, Currie KD, Nouraei H, Sadeghi M, Krassioukov AV. Multi-domain assessment of autonomic function in spinal cord injury using a modified autonomic reflex screen. Journal of Neurotrauma. 2017 9 15;34(18):2624–33. 10.1089/neu.2016.4888 28537464

[12] Monahan KD, Dinenno FA, Tanaka H, Clevenger CM, DeSouza CA, Seals DR. Regular aerobic exercise modulates age‐associated declines in cardiovagal baroreflex sensitivity in healthy men. The Journal of physiology. 2000 11;529(1):263–71. 10.1111/j.1469-7793.2000.00263.x 11080267 PMC2270167

[13] Caminiti G, Volterrani M, Iellamo F, Marazzi G, Massaro R, Miceli M, et al. Effect of long-acting testosterone treatment on functional exercise capacity, skeletal muscle performance, insulin resistance, and baroreflex sensitivity in elderly patients with chronic heart failure: a double-blind, placebo-controlled, randomized study. Journal of the American College of Cardiology. 2009 9 1;54(10):919–27. 10.1016/j.jacc.2009.04.078 19712802

[14] Kirshblum S, Waring W. Updates for the international standards for neurological classification of spinal cord injury. Physical Medicine and Rehabilitation Clinics. 2014 8 1;25(3):505–17. 10.1016/j.pmr.2014.04.001 25064785

[15] Shimada KA, Kitazumi TA, Sadakane NO, Ogura HI, Ozawa TO. Age-related changes of baroreflex function, plasma norepinephrine, and blood pressure. Hypertension. 1985 1;7(1):113–7. 10.1161/01.hyp.7.1.113 3980053

[16] American College of Sports Medicine. ACSM’s guidelines for exercise testing and prescription. Lippincott Williams & Wilkins; 2013 3 4. 10.1249/JSR.0b013e31829a68cf 23851406

[17] Taylor JA, Picard G, Widrick JJ. Aerobic capacity with hybrid FES rowing in spinal cord injury: comparison with arms-only exercise and preliminary findings with regular training. PM&R. 2011 9 1;3(9):817–24. 10.1016/j.pmrj.2011.03.020 21944299

[18] Goldstein DS, Horwitz D, Keiser HR. 1982. Comparison of techniques for measuring baroreflex sensitivity in man. Circulation. 1982 8;66(2):432–9. 10.1161/01.cir.66.2.432 7094250

[19] Parati G, Di Rienzo M, Mancia G. How to measure baroreflex sensitivity: from the cardiovascular laboratory to daily life. Journal of hypertension. 2000 1 1;18(1):7–19. 10678538

[20] Smyth HS, Sleight P, PICKERING GW. Reflex regulation of arterial pressure during sleep in man: a quantitative method of assessing baroreflex sensitivity. Circulation research. 1969 1;24(1):109–21. 10.1161/01.res.24.1.109 4303309

[21] Eckberg DL, Mohanty SK, Raczkowska M. Trigeminal‐baroreceptor reflex interactions modulate human cardiac vagal efferent activity. The Journal of physiology. 1984 2 1;347(1):75–83. 10.1113/jphysiol.1984.sp015054 6707976 PMC1199435

[22] Coupe M, Fortrat JO, Larina I, Gauquelin-Koch G, Gharib C, Custaud MA. Cardiovascular deconditioning: from autonomic nervous system to microvascular dysfunctions. Respiratory physiology & neurobiology. 2009 10 1;169:S10–2. 10.1016/j.resp.2009.04.009 19379845

[23] Kaushal P, Taylor JA. Inter-relations among declines in arterial distensibility, baroreflex function and respiratory sinus arrhythmia. Journal of the American College of Cardiology. 2002 5 1;39(9):1524–30. 10.1016/s0735-1097(02)01787-4 11985918

[24] Kelley GA, Kelley KA, Vu Tran Z. Aerobic exercise and resting blood pressure: a meta‐analytic review of randomized, controlled trials. Preventive cardiology. 2001 4;4(2):73–80. 10.1111/j.1520-037x.2001.00529.x 11828203 PMC2094526

[25] Hubli M, Krassioukov AV. Ambulatory blood pressure monitoring in spinal cord injury: clinical practicability. Journal of neurotrauma. 2014 5 1;31(9):789–97. 10.1089/neu.2013.3148 24175653 PMC3997095

[26] Solinsky R, Vivodtzev I, Hamner JW, Taylor JA. The effect of heart rate variability on blood pressure is augmented in spinal cord injury and is unaltered by exercise training. Clinical Autonomic Research. 2020 3 12:1–9. 10.1007/s10286-020-00677-2 32166421 PMC9270103

[27] Alan N, Ramer LM, Inskip JA, Golbidi S, Ramer MS, Laher I, Krassioukov AV. Recurrent autonomic dysreflexia exacerbates vascular dysfunction after spinal cord injury. The Spine Journal. 2010 12 1;10(12):1108–17. 10.1016/j.spinee.2010.09.018 21094471

[28] Jegede AB, Rosado-Rivera D, Bauman WA, Cardozo CP, Sano M, Moyer JM, et al. Cognitive performance in hypotensive persons with spinal cord injury. Clinical Autonomic Research. 2010 Feb 1;20(1):3–9. 10.1007/s10286-009-0036-z PMC2899478 19842013

[29] Brown R, Stolzenhein G, Engel S, Macefield VG. Cutaneous vasoconstriction as a measure of incipient autonomic dysreflexia during penile vibratory stimulation in spinal cord injury. Spinal Cord. 2009 7;47(7):538–44. 10.1038/sc.2008.158 19079355

[30] Wecht JM, Bauman WA. Implication of altered autonomic control for orthostatic tolerance in SCI. Autonomic Neuroscience. 2018 1 1;209:51–8. 10.1016/j.autneu.2017.04.004 28499865

[31] Solinsky R, Svircev JN, James JJ, Burns SP, Bunnell AE. A retrospective review of safety using a nursing driven protocol for autonomic dysreflexia in patients with spinal cord injuries. The journal of spinal cord medicine. 2016 11 1;39(6):713–9. 10.1080/10790268.2015.1118186 26838482 PMC5137561

[32] Morady FR, Kou WH, Nelson SD, de Buitleir MI, Schmaltz ST, Kadish AH, et al. Accentuated antagonism between beta-adrenergic and vagal effects on ventricular refractoriness in humans. Circulation. 1988 2;77(2):289–97. 10.1161/01.cir.77.2.289 3338125

