# Supplementary material for: High-intensity, whole-body exercise improves blood pressure control in individuals with spinal cord injury: A prospective randomized controlled trial
Source: PLoS One. 2021 Mar 4;16(3):e0247576. doi: 10.1371/journal.pone.0247576 (PMC7932070; doi:10.1371/journal.pone.0247576)
Supplement: S1 File — (DOCX) [file pone.0247576.s002.docx]

**PROTOCOL TITLE**:

Hybrid-FES Exercise to Prevent Cardiovascular Declines in Acute and Chronic SCI

**I. SUBJECT SELECTION**

We will enroll 60 individuals within twelve months post SCI to obtain data on 50 individuals. One-half of the subjects (N=25) will be randomized to immediately enroll in 6 months of FES-RT. One third (N=18) will be randomized to a wait-list to provide time control data from baseline to 6 months. A wait-list control group is routine in exercise studies because most volunteers are interested in participating in an exercise program. Hence, time controls are difficult to capture since many of those randomized to receive no exercise either drop from the study entirely, or end up pursuing some form of exercise on their own. Therefore, a vehicle for enrollment of a time control group that is acceptable to most volunteers is a wait-list. A six month time control will provide data on expected declines. One-third (N=17) of subjects will be randomized to six months of arms-only-RT. This will provide data on a comparator that represents a form of arms-only exercise that would be available to those with SCI. These subjects will undergo six months of FES-RT after completing arms-only-RT. This will enhance subject retention and provide data for secondary analysis.

To enhance subject recruitment we may enroll subjects into the study before they are 3-months post injury, most likely while they are still in-patients at Spaulding Rehabilitation Hospital. This will help subjects to schedule and plan for transportation for study assessments and training to begin when they reach 3-months post injury. No study assessments will be scheduled before 3-months from time of their SCI. Study participation will not interfere with scheduling of any normal inpatient therapy, nor will study participation take place during normal therapy. In-patients will need to be cleared to participate by their treating physician.

In addition we will enroll another 25 individuals with chronic SCI to serve as a comparator to the acute FES-RT group. Neither a wait-list nor arms-only-RT are required for this population. Time control data from baseline to 6 months are not needed since expected declines would be minimal over this time period after the acute phase of injury. In addition, the fourth aim is solely focused on responses to FES-RT and so a comparator that represents a form of arms-only exercise available to those with SCI is not necessary. Subjects in the chronic group will be at least 1 year post spinal cord injury with adult onset of SCI(SCI occurrence after 18 years of age).

All volunteers will continue with regular physical rehabilitation except for modalities that overlap study interventions (e.g., FES-cycling).

All Subjects will be 18-40 years of age with spinal cord injury (American Spinal Injury Association A, B, C) at the neurological level of C5-T12. Subjects will be medically stable (no treatment for DVT, no orthostasis, no spinal precautions or weight-bearing precautions associated with a healing long-bone fracture) and be able to tolerate the stimulation without dysreflexia. Dysreflexia in some participants is expected in the initial FES sessions, but only those in whom dysreflexia abates with repeat exposures will be included.

Participants will be excluded if they have: 1)Hypertension (blood pressure >140/90 mmHg), 2) significant arrhythmias, 3) coronary disease, 4) diabetes, 5) renal disease, 6) cancer, 7) epilepsy, 8) current use of cardioactive or antidepressant medications, 9) family history of significant arrhythmia or sudden cardiac death 10) orthostatic hypertension with symptomatic fall in blood pressure > 30 mmHg when upright 11) current grade 2 or greater pressure ulcers at relevant contact sites, 12) other neurological disease, 13) peripheral nerve compressions or rotator cuff tears that limit the ability to row, 14) history of bleeding disorder, 15) regular use of tobacco,

16) current deep venous thrombosis, 17) severe spasticity (that prevent rowing movements), 18) severe autonomic dysreflexia, 19) implanted electronic cardiac device (pace maker, cardiac defibrillator), 20) ventilator-dependent condition, 21) sensations that prevent the use of the electrical stimulation, 22) other medical contraindications to vigorous exercise.

The American College of Sports Medicine’s Guidelines for Exercise Testing and Prescription 9^th^ edition will be followed as the standard of care along with the clinical specialists’ opinion (Drs. Stephanie Cho and Aaron Baggish) regarding any additional pre-participation screening needed prior to beginning this vigorous exercise training program.

Pregnant and nursing women are not eligible for this study because of the potential risk to the fetus from radiation that goes along with the DXA scan. Women who are able to become pregnant must agree to use contraceptive methods while participating in the study. Acceptable contraceptive methods are: a) hormonal methods (birth control pills, patches, injections, vaginal ring or implants); b) barrier methods (condom or diaphragm, with a foam, cream or gel spermicide); c) intrauterine device (IUD); or d) abstinence (no sex). Women are considered unable to become pregnant if they are menopausal and have not had a menstrual period for at least one year or if they have had a hysterectomy (surgical removal of your uterus and/or ovaries).

**II. SUBJECT ENROLLMENT**

Research study staff will obtain volunteer consent in accordance with guidelines established by the Institutional Review Board during the first visit to the Laboratory. Participants will be sent the consent form at least 48 hours in advance so they have ample time to read and ask questions. Participants are encouraged to ask questions and are reminded that participation is strictly voluntary and will not affect their current or future care at Spaulding Rehabilitation Hospital or any of its affiliates.

**III. STUDY PROCEDURES**

Volunteers who enroll in the study before 3-months from their injury date will have a partial screening visit in which informed consent and detailed health history will be obtained(all other assessments will be scheduled after they reach at least 3-month post SCI. Once recruited for the study volunteers (that are at least 3-months post injury) will visit the laboratory for a health screening session to further determine eligibility. This two may require one or two visits depending on the ability to schedule with the physician. It will include obtaining informed consent, detailed health history, PROMIS 57 questionnaire, a standard physical exam (ASIA exam and Ashworth Scale) performed by a physician (if necessary), height and weight measurements, resting blood pressure, electrocardiogram (EKG) and FES-device test**.**

Note: If an ASIA exam and Ashworth Scale have previously been performed by a physician and results can be obtained, those medical records may be used and the physical exam will not be necessary during screening.

Subjects may also be asked to provide additional medical records or may need further testing to determine their eligibility and the safety of their participation in this program which includes regular vigorous exercise. These additional tests may or may not be covered by insurance. The subject will be responsible for obtaining any additional testing and providing the program with medical records or results.

Approximately 40% of spinal cord injured women have amenorrhea for an average of eight months. Therefore, menstrual status will be tracked and explored as a potential covariate. A prospective menstrual diary will be collected for three months prior to each data acquisition time-point. For each monthly diary, women will mark the day of the month when bleeding occurred and rate the heaviness of blood flow (spotting to heavy). If no bleeding occurred during a particular month or the menstrual period, this will be indicated. Bleeding patterns will be used to define normal, irregular, or absent menstrual cycles.

**Training Protocols for Each Study Group**

**1) FES-RT GROUP (Acute and Chronic SCI)**

FES-strength training prior to FES-RT

To be able to FES-row, a certain level of leg muscle strength and endurance is essential; therefore, prior to FES-row training, the ability of the subject to perform 30 minutes of FES-induced leg extension will be evaluated. Electrodes will be placed over the motor points of the rectus femoris, vastus medialis, vastus lateralis, biceps femoris and semitendinosos . A four-channel Odstock stimulator will alternate stimulation between the hamstrings and the quadriceps while the subject is seated in their wheelchair or on an exam table. The stimulus parameters will be set at 12 second periods with a 6 second on-time per channel without a ramp and with a pulse width of 450ms at 50 Hz. The intensity of the stimulus to the quadriceps will be set at the level that produces full knee extension. If the knee does not achieve full extension due to fatigue the intensity of the stimulation will be increased. If fatigue does not occur prior to 30 minutes, the individual will advance to FES-rowing. If fatigue occurs prior to 30 minutes and at maximal intensity (approximately 100mA), the subject will engage in two weeks (3x/week) of strength training using this same protocol. Previous experience has indicated that this protocol is effective in enhancing muscle strength. Following two weeks of training, the strength and endurance capacity of the knee-extensor muscles will be evaluated and further training will be performed, if necessary, including the additional use of the QAA-9054 Total Power strength training machine. The QAA-9054 is a unique machine that provides the safety of accommodating resistance without the need of eccentric contractions. This type of resistance will not only allow the proper resistance to be safely placed on the knee extensors but also the knee flexors. Our past experience has shown that the knee flexors may lag behind the knee extensors and prevent continuous FES-rowing. The Strength training will continue until the subject is able to complete the knee flexion-extension protocol for 30 minutes without rest. This may take between 2 and 12 weeks depending on the subject’s response to the FES-strength training. Subjects will also be encouraged to begin arms only rowing during the initial FES-strength training period. This will be done at the same visits as the FES-strength training sessions. Participants will be seated on the rowing machine with the rowing seat locked in a fixed position and will perform short intervals of rowing at a moderate intensity using only their arms. Intensity will be monitored by heart rate and rating of perceived exertion. Subjects will be allowed to take the FES-device home after they have completed the first two weeks of training and have shown adequate understanding of how to use the device properly. Subjects will perform strength training session at Spaulding Boston or Spaulding Cambridge as needed during home strength training. Subjects will also be given a logbook to track their usage.

### FES-RT

### Subjects will begin with short intervals of FES-RT interspersed with rest intervals and/or arms-only rowing intervals depending on fitness level and the response to the FES. A maximum FES-rowing test will be performed at baseline. This will be repeated after three months of training and training intensity will be adjusted to maintain the training stimulus at the same relative intensity. The goal is for each volunteer to achieve an exercise intensity of 75-85% maintained for a continuous 30 minutes performed three times each week. A maximum FES-rowing test will be performed at the end of the six months of FES-RT to determine increases in fitness. Measurements of force produced at the foot and handle during FES-row training sessions will also be used to monitor training and possibly improve rowing technique.

###### Strength Training during FES-RT

After the first two weeks of FES-leg strength training (it may take longer depending on the subject’s response, see above) and when the subjects have demonstrated the ability to properly use the stimulation unit, the stimulation unit will be used at home to continue with leg strengthening on non FES-rowing days (~3 days/week, 30-60 minutes/session). This can be closely monitored since they will also be exercising 3 times per week at Spaulding Boston or Spaulding Cambridge.

**2) ARMS-ONLY-RT GROUP(Acute SCI only)**

### The arms-only-RT group will commence exercise training upon enrollment. Training sessions will be 3 times per week for 26 weeks. To parallel the FES-RT, the initial training sessions will also consist of 6 sets of arms-only rowing for five minutes at 60% of VO2peak with a work-to-rest ratio of 2:1 and progress over the six months to an exercise intensity of 75-85% maintained for 30 minutes performed three times each week. The arms-only-RT group will also perform the FES-leg strength training protocol as described above during their six months of arms-only training. During this time they will not use the FES-device on the rowing ergometer. Strengthening the leg muscles during the arms-only period will lessen leg muscle atrophy and allow faster progress in FES-row training when they enter that phase of the study. A maximum arms-only rowing test will be performed at baseline. This will be repeated after three months of training and training intensity will be adjusted to maintain the training stimulus at the same relative intensity. A maximum arms-only rowing test will be performed at the end of the six months of training to determine increases in fitness. Subsequently, after the six months of arms-only-RT, subjects will begin the FES-RT as described above.

**3) WAIT-LIST GROUP(Acute SCI only)**

The wait list group will not participate in any row training for 6 months. A maximum arms-only rowing test will be performed immediately after enrollment, after initial familiarization with arms-only-RT equipment (usually 2-3 sessions) and will be repeated after 3 and 6 months (before FES-RT begins). The wait-list group will also perform the FES-leg strength training protocol as described above during the six months of the wait-list period. During this time they will not use the FES-device on the rowing ergometer. Strengthening the leg muscles during the wait-list period will lessen leg muscle atrophy and allow faster progress in FES-row training when they enter that phase of the study.

At the end of the 6 month wait period, subjects will begin the FES-RT as described above.

Notes: There is no limit or specific requirement for training sessions that must be attended to continue participating in this protocol. Subjects, in all training groups, will be encouraged by study staff to attend three training sessions per week. Un-reported missed training session will be followed up with a phone call to document why the training session was missed and to encourage prompt return to training sessions as soon as possible.

Subjects who enroll before 3-months from their date of SCI will be given the opportunity to participate in the FES-strength training during the period before study assessments begin after they reach 3-months post SCI. This will lessen leg muscle atrophy and allow faster progress in FES-row training when they enter that phase of the study.

**Study Assessments**

Most Assessments will be performed at baseline and after 3 and 6 months for each study group. The DXA measurements and the AO-VO2max test (for the FES-RT group only) will only be performed at baseline and after 6 months. The Arms-only-RT and Wait-list groups will immediately enter baseline FES-RT protocol after completing all study procedures required in their initial group. Their six month lab visit and DXA scan data will also be used as their baseline FES-RT measures. Subjects may be asked to repeat any of the study assessments if there is difficulty obtaining results that meet our quality standards or there are equipment issues that arise.

Subjects that take longer than eight weeks to progress to performing their baseline FES-VO2max test will perform an extra assessment around the time of their baseline FES-VO2max test to account for changes that may occur over that time period. This extra study session will consist of: height, weight and circumference measures, blood samples, health questionnaires, pulmonary function tests, resting cardiovascular hemodynamics and neck suction-barorefex testing. This extra testing session will take about 2-hours and can be done on a normal training day.

Study Procedure Time Line:

|  | Baseline | 3-Month | 6-Month | Baseline-RT | 3-Month-RT | 6- Month-RT |
| --- | --- | --- | --- | --- | --- | --- |
| FES-RT  Acute and Chronic | FES-VO2max  AO-VO2max  Lab Visit  DXA Scan | FES-VO2max  Lab Visit | FES-VO2max  AO-VO2max  Lab Visit  DXA Scan | N/A | N/A | N/A |
| AO-RT  Acute | AO-VO2max  Lab Visit  DXA Scan | AO-VO2max  Lab Visit | AO-VO2max  Lab Visit  DXA Scan | FES-VO2max | FES-VO2max  Lab Visit | FES-VO2max  Lab Visit  DXA Scan |
| Wait list  Acute | AO-Vo2max  Lab Visit  DXA Scan | AO-VO2max  Lab Visit | AO-VO2max  Lab Visit  DXA Scan | FES-VO2max | FES-VO2max  Lab Visit | FES-VO2max  Lab Visit  DXA Scan |

**Measurements and Testing Protocols:**

VO2max Testing

For subjects in the arms-only-RT and FES-RT groups VO2max testing will be performed on a day scheduled for regular training. On-line computer-assisted open circuit spirometry will be used to determine O_2_ consumption, CO_2_ production, and respiratory exchange ratio. Expired O_2_ and CO_2_ gas fractions will be measured with a paramagnetic O_2_ and infrared CO_2_ analyzers. Ventilation will be measured via a Hans Rudolph 3813 pneumotachograph. Subjects will perform FES-rowing or arms-only-rowing while increasing power output every 1 to 2 minutes until volitional exhaustion. Immediately after FES-rowing or arms-only-rowing has stopped at the end of the VO2max test, subjects will have a finger tip pricked with a safety lancet in order to get a drop of blood that will be used to measure peak lactate levels in whole blood. Peak lactate levels will be used to evaluate exercise intensity as well as to monitor training status. To ensure attainment of maximal exercise capacity, at least 3 of the following criteria will be met: 1) O_2_ consumption plateaus despite increasing workload, 2) respiratory exchange ratio equals or exceeds 1.10 at end exercise, 3) 85% age-predicted maximal heart rate is achieved, and 4) perceived exertion is rated at least 17 on the Borg scale of 6-20.

Lab Visit(s)

All lab studies will be performed in the morning. All subjects will be instructed to abstain from vigorous exercise for 2 days prior to each study to avoid autonomic and neuroendocrine effects of exercise. In addition, subjects will refrain from caffeine and alcohol for the previous 24 hrs, and be studied after a 12 hr fast.

Neck Suction- Baroreflex Testing:

We will rely on neck pressure and neck suction to evaluate carotid sinus baroreceptor function at rest and during a variety of conditions. This technique has been employed to safely study cardiovascular physiology in human since the 1950’s. The subject’s neck is sealed by a neck collar connected to a computer-controlled bellows (pressure/vacuum pump). The neck collar is cushioned with an airtight silicon rubber bladder that is independently sealed and positioned against the contour of the anterior lateral neck. The bellows is used to increase or decrease pressure inside the neck collar, effectively compressing or distending the carotid sinus in the neck and causing reflex changes in heart rate and muscle sympathetic activity. However, these responses are evanescent since aortic baroreceptors are not affected; therefore heart rate and sympathetic activity (and sometimes blood pressure) are changed for only a few beats with any level of stimulus. Carotid baroreceptor reflex responses will be elicited during held expiration by application of an external neck pressure and suction sequence. Subjects will hold a normal end-expiratory volume, which triggers an external neck pressure increase of +40 mmHg for 4 heart beats and subsequent, serial R-wave triggered reductions of 15 mmHg to a pressure of -65 mmHg. This sequence results in stepwise initial carotid sinus compression followed by distension, evoking stepwise reflex changes in heart rate and sympathetic activity. For each baroreflex testing, this procedure is repeated seven times to provide an averaged reproducible carotid baroreceptor reflex response relation.

*Echocardiography.* We will use a portable, commercially available system (Vivid-I, GE Healthcare, Milwaukee, Wis.), for cardiac characterization. Images will be obtained after 20 minutes of rest. Conventional 2-dimensional, Doppler, and color tissue-Doppler imaging from standard parasternal, apical, and subcostal positions will be performed. All data will be stored digitally for post-study off-line data analysis (EchoPac, Version 6.5, GE Healthcare). Left ventricular and atrial cardiac chamber volumes will be calculated using the modified Simpsons technique. LV mass will be calculated using the area-length method. Relative wall thickness, an index of LV geometry, will be defined as: [interventricular septal thickness (mm) + posterior wall thickness (mm)] / LV internal end-diastolic diameter (mm). Myocardial tissue velocities (diastolic function) will be measured off-line from 2D color-coded tissue Doppler images and reported as the average of 3 consecutive cardiac cycles. Strain, strain rate, and LV rotational (systolic function) assessment will be performed by speckled tracking analysis. Peak global strain will be calculated as the average strain in the basal, mid, and apical LV as measured in the apical 4-chamber view. Atrial volumes will be calculated using the bi-plane area-length method. Right ventricular diastolic area, systolic area, fractional area change, tissue velocities will be measured. Measurements will be indexed for body surface area when appropriate.

Note: Subjects may also have their echocardiogram test performed at the Heart Center at Massachusetts General Hospital (MGH) if the cardiologist can be scheduled for a more convenient time for them at MGH than at Spaulding Cambridge. We will pay for parking if they choose to go to MGH for their echocardiogram.

Note: As of 1/1/19 we will no longer be able to measure any further echocardiogram tests in this protocol.

Resting Cardiovascular Hemodynamics: Heart rate and blood pressures will be measured after a 15-minute period of quiet supine rest. Heart rate will be measured by a standard 12-lead ECG and blood pressure by an automated sphygmomanometer. There will be a short period of time paced breathing in which subjects follow an audio cue to breath at a rate of 15 breaths per minute.

Pulmonary Function Tests: Spirometry will be used to measure lung function, specifically the measurement of the amount (volume) and/or speed (flow) of air that can be inhaled and exhaled. Specifically, Forced Volume Vital Capacity (FVC) and Maximum Voluntary Ventilation (MVV) tests will be administered.

Height, Weight, and Circumferences: Weight will be measured on a scale. Height will be measured in the supine position with a tape measure. Circumferences of the waist, hip, abdomen, and thigh will be measured with a tape measure in the supine position.

Blood Samples: 70 ml of blood via venipuncture will be taken. Standard assays will include measures of insulin sensitivity and serum lipids. The homeostasis model assessment (HOMA) of insulin resistance, the quantitative insulin check index (QUICKI) and the serum insulin-like growth factor binding protein-1 (IGFBP-1) will be assessed. Plasma total cholesterol, low density cholesterol, high density APOa lipids, and triglycerides will also be measured. Other assays will include: total testosterone, calculated free testosterone, CBC, high sensitivity (hs) C-reactive protein, hemoglobin AIC, prealbumin and zinc. Blood work will be sent to Quest Diagnostics for analysis.

ASIA and Modified Ashworth exams: Standard physical exams will be performed by a physician to categorize injury level and degree of spasticity.

Note: The ASIA and Modified Ashworth exams will only be performed at Screening Visit 1 and 6-month and 12-month time points.

PROMIS 57 Questionnaire: The PROMIS 57 questionnaire will be used to assess changes in subject’s views on their physical, psychological, and social well-being. The PROMIS 57 scales include eight items from seven primary PROMIS domains: depression, anxiety, physical function, pain interference (and one pain intensity item), fatigue, sleep disturbance and satisfaction with social roles.

PROMIS Cognition Short Form: a brief well validated self-report measure to assess perceived cognitive functioning.

The Patient Health Questionnaire (PHQ-9): a brief, well validated depression screening test that is widely used in medical research.

NIH Toolbox Meaning and Purpose Short Form: is a 7 item questionnaire that measures a person’s sense of meaning and purpose in life over the past 7 days.

Craig Handicap Assessment and Reporting Technique Short Form (CHART-SF): is valid, reliable, and widely used in rehabilitation research to measure various community integration in people with various neurological conditions. We will be collecting data on the Physical Independence, Mobility, Occupation, and Social Integration subscales.

SCI Exercise Self- Efficacy Scale (ESES): is a reliable and valid measurement of self-efficacy, or belief and confidence in one's skill and ability, to engage in physical activity.

DXA Scan(s)

A licensed radiology technician will perform all DXA scans. We will use a 5th generation GE Healthcare iDXA dual x-ray absorptiometry (DXA) scanner with enCore configuration version 12.3. For regional fat measurements, the DXA software can be used to define standard regions that will allow comparability of measurements throughout the study. DXA-derived visceral adipose tissue will be measured from a 5 cm slice placed across the entire abdomen above the iliac crest at the 4th lumbar vertebrae. Although DXA measures both visceral and subcutaneous fat, on each side of the abdominal cavity DXA can directly measure subcutaneous fat. The location of the abdominal cavity is detected by the gray scale change as the tissue contains lesser fat due to abdominal wall muscles. Subcutaneous fat over the visceral cavity is estimated from the DXA measurement of subcutaneous fat on each side of the abdominal cavity. The total estimated subcutaneous fat is subtracted from the total abdominal fat for DXA-derived visceral adipose tissue. This approach has been shown to performed as well as a clinical read from a CT scan. The precision of regional fat measures is ±1%.

Note: If a DXA scan has recently been performed at Spaulding Rehabilitation Hospital and the results can be obtained, those medical records may be used and the baseline DXA scan will not be necessary.

**IV. BIOSTATISTICAL ANALYSIS**

*Power analysis:* Based on longitudinal exercise capacity data from our lab, extensive sets of power analyses were performed. For the main analysis (acute subjects only), there will be 3 groups of subjects (FES-RT, wait-list and arms-only-RT) and measures at baseline, 3 months, 6 months, except for visceral adiposity (only at baseline and 6 months). The primary analysis (below) will include the three groups and will have as predictors two indicator (dummy) variables for two of the groups, and indicators for the 3 month and 6 month time points and interactions between the time points and the group indicator variables; this will be estimated as a linear mixed model. The only random effect will be for subject. In addition, for the fourth aim, the analysis will only include those entered into into FES-RT at the outset, i.e., the FES-RT with acute SCI and all volunteers with chronic SCI. The main analysis will consist of comparisons across time with injury duration as a covariate.

Given logistical constraints, we began with the assumption that data on 50 acute SCI subjects (out of 60 recruited - a ~15% dropout rate) would be advantageous if that sample size allowed us to find differences that are substantively meaningful. This group would be allocated (randomly; see below) as 40% to FES-RT and 30% to each of wait-list and arms-only-RT. We also checked other total sample sizes and allocation rules (e.g., data from 20 in each group, data from 25 in FES and 15 in each of the other two groups). In addition, 25 individuals with chronic SCI would provide sufficient power to detect meaningful (e.g., >15%) differences between acute and chronic SCI at the same power outlined below.

These calculations were performed assuming an alpha of 0.05 and a correlation between baseline and 6 month measure of about 0.7; unless the correlation was very different from 0.7 (e.g., 0.25), there was little difference in the results. This shows that we will have sufficient power under this plan with a sample size of 50 acute SCI subjects and allocation of 20/15/15. Subjects will be randomized at the ratio of 1.33:1:1 to each of the three groups by a randomization plan constructed by the project statistician. The investigator will have no knowledge of group assignment until the time of subject enrollment. During the study, there can be no blinding of the subject or the investigator, however all measures will be obtained by an individual blind to subject group assignment.

*Primary data analysis:* The primary analysis will test whether the FES-RT has ‘better’ values of the outcome variables at 6 months post-baseline. A general linear mixed model with a random effect for subjects will be used to conduct an intent-to-treat analysis. The main predictors will be indicator variables for group with the FES-RT group as the reference group and indicators for each of the other two groups, the time at which the outcomes were measured and treatment group by time interactions; menstrual status (with men coded the same as women who are not menstruating) will be included as a dummy variable; for the insulin resistance measure, body weight/fat changes will be included as a predictor. In a secondary analysis, we will include interactions terms between the group indicators and the 3 month indicator. In addition, similar models to the above will be estimated for secondary outcomes.

*Checking and Sensitivity Analyses:* It is important to ensure that data idiosyncrasies do not impact the results and that the results are generalizable. We will check for systematic differences between the model and the data using graphs, such as comparisons of predicted and observed values of the outcome variable, and other standard diagnostics. An extension of this idea is to simulate new sets of data based on our model and use the simulated data as a reference test group by comparing this to the observed data; we do this to look for situations in which the data appear different from what we would expect by using the model to predict the data. We will also check whether any important predictors have been left out of the model, for example, gender or severity of SCI or level of SCI. These will be tested by including them in revised versions of the model and testing whether the model is substantively improved. It is also important to explore the ‘missingness’ of data. The model we plan to use assumes that missing data are “missing at random.” This can be checked in several ways including comparing the characteristics of observations with and without missing values. More importantly, we can check whether our results would change if the data is “missing not at random” in several ways. For certain outcome variables, it may be the case that subjects who are either at the high end or the low end are more likely to be missing. For example, people at baseline who are very fat or very lean may be more likely to drop out. This can be checked by using constrained multiple imputation where we constrain our imputations to one end of the adiposity range. Comparing the results based on the constrained imputation to the unimputed results gives us a sensitivity analysis.

A second type of sensitivity analysis was originally suggested by Rubin and has been extended by Carpenter, Kenward and White who suggest weighting each imputed result (rather than Rubin's standard “rules” of averaging the results), where the weight depends on the assumed departure from the MAR assumption. This technique relies on the imputations covering a fairly wide range of values (we would use 100 imputations) but this can be checked and additional data sets imputed if the range is too narrow. Carpenter, *et al.,* provide a graphic method of checking whether the range is wide enough. Finally, if dropouts are much more common that missing an occasion and then returning, we will estimate a pattern-mixture model. However, if there are many people who miss the 3 month measure, but not the 6 month measure, we will not pursue this strategy. A fourth issue deals with the variance structure of the model. Our prime model assumes that the variances are the same across groups. This will be tested by estimating a model with heterogeneous variance structures by group (this is available in some statistical packages, including Stata or SAS).

**X. REFERENCES**

Reference List

(1) Myers J, Lee M, Kiratli J. Cardiovascular disease in spinal cord injury: an overview of prevalence, risk, evaluation, and management. *Am J Phys Med Rehabil* 2007;86:142-152.

(2) Cragg JJ, Stone JA, Krassioukov AV. Management of cardiovascular disease risk factors in individuals with chronic spinal cord injury: an evidence-based review. *J Neurotrauma* 2012;29:1999-2012.

(3) Galea MP. Spinal cord injury and physical activity: preservation of the body. *Spinal Cord* 2012;50:344-351.

(4) Taylor JA, Picard G, Widrick JJ. Aerobic capacity with hybrid FES rowing in spinal cord injury: comparison with arms-only exercise and preliminary findings with regular training. *PM R* 2011;3:817-824.
