# Supplementary material for: High-intensity, whole-body exercise improves blood pressure control in individuals with spinal cord injury: A prospective randomized controlled trial
Source: PLoS One. 2021 Mar 4;16(3):e0247576. doi: 10.1371/journal.pone.0247576 (PMC7932070; doi:10.1371/journal.pone.0247576)
Supplement: S2 File — (DOCX) [file pone.0247576.s003.docx]

**S3. Full data available at *Harvard dataverse*** https://doi.org/10.7910/DVN/JJGTYM
